# Evidence of exposure to *C. burnetii* among slaughterhouse workers in western Kenya

**DOI:** 10.1016/j.onehlt.2021.100305

**Published:** 2021-08-10

**Authors:** Elizabeth Anne Jessie Cook, William Anson de Glanville, Lian Francesca Thomas, Alice Kiyong'a, Velma Kivali, Samuel Kariuki, Barend Mark de Clare Bronsvoort, Eric Maurice Fèvre

**Affiliations:** a International Livestock Research Institute, Old Naivasha Road, PO Box 30709, 00100 Nairobi, Kenya; b Center for One Health, University of Global Health Equity, Rwanda; c Institute of Infection, Veterinary and Ecological Sciences, University of Liverpool, Leahurst Campus, Chester High Road, Neston CH64 7TE, UK; d Kenya Medical Research Institute, PO Box 19464-00200, Nairobi, Kenya; e Roslin Institute, The Royal (Dick) School of Veterinary Studies, University of Edinburgh, Roslin, Midlothian EH25 9RG, UK; f Royal (Dick) School of Veterinary Studies, University of Edinburgh, Roslin, Midlothian EH25 9RG, UK

**Keywords:** Slaughterhouse, Kenya, Q fever, *C. burnetii*, Zoonoses, Occupational health

## Abstract

Q fever, caused by *C. burnetii*, has been reported in slaughterhouse workers worldwide. The most reported risk factor for seropositivity is the workers' role in the slaughterhouse. This study examined the seroprevalence and risk factors for antibodies to *C. burnetii* in slaughterhouse workers in western Kenya to fill a data gap relating to this emerging disease in East Africa.

Individuals were recruited from all consenting slaughterhouses in the study area between February and November 2012. Information was collected from participating workers regarding demographic data, animals slaughtered and role in the slaughterhouse. Sera samples were screened for antibodies to *C. burnetii* using a commercial ELISA and risk factors associated with seropositivity were identified using multi-level logistic regression analysis.

Slaughterhouse workers (*n* = 566) were recruited from 84 ruminant slaughterhouses in western Kenya. The seroprevalence of antibodies to *C. burnetii* was 37.1% (95% Confidence Interval (CI) 33.2–41.2%). The risk factors identified for *C. burnetii* seropositivity included: male workers compared to female workers, odds ratio (OR) 5.40 (95% CI 1.38–21.22); slaughtering cattle and small ruminants compared to those who only slaughtered cattle, OR 1.52 (95% CI 1.06–2.19). In addition, specific roles in the slaughterhouse were associated with increased odds of being seropositive, including cleaning the slaughterhouse, OR 3.98 (95% CI 1.39–11.43); cleaning the intestines, OR 3.24 (95% CI 1.36–7.73); and flaying the carcass OR 2.63 (95% CI 1.46–4.75) compared to being the slaughterman or foreman.

We identified that slaughterhouse workers have a higher seroprevalence of antibodies to *C. burnetii* compared to published values in the general population from the same area. Slaughterhouse workers therefore represent an occupational risk group in this East African setting. Workers with increased contact with the viscera and fluids are at higher risk for exposure to *C. burnetii*. Education of workers may reduce transmission, but an alternative approach may be to consider the benefits of vaccination in high-risk groups.

## 1 Introduction

*C. burnetii* is the causative agent of Q fever, an underreported zoonotic disease [1]. The disease was first recognised in slaughterhouse workers in Australia in 1937 [2] and outbreaks have since been reported worldwide [1]. The biggest reported human Q fever outbreak occurred between 2007 and 2010 in the Netherlands. The outbreak involved at least 4000 human cases and infections were associated with proximity to infected goat farms [3].

The primary reservoirs for human infection are ruminants including cattle, sheep, and goats [4]. The bacterium is shed by infected animals in excreta including faeces, urine, milk, and placental fluids [1]. Transmission is through direct contact with animal fluids, ingestion of milk, or aerosols from materials contaminated by infected animals [5]. The bacterium persists in the environment and has a very low infectious dose; one bacterium can result in infection [5].

Human infections may be asymptomatic, or develop into a mild, nonspecific flu-like illness [1]. However, more serious sequelae such as endocarditis may result from untreated chronic Q fever [6]. The presenting signs are typical of a febrile illness including fever, headaches, chills, and sweating. Other signs include: coughing, joint and muscle pain, atypical pneumonia, and hepatitis [7]. The reference technique for the diagnosis of Q fever is immunofluorescence antibody. [8,9]. The recommended test for seroprevalence studies is the IgG Phase 2 ELISA since antibodies can be present for over 5 years [9,10].

Q fever outbreaks have been documented in multiple countries with slaughterhouse workers considered a high risk group [11,12]. The primary risk factor identified for seropositivity to *C. burnetii* in slaughterhouse workers is the role within the slaughterhouse particularly those workers exposed to hides and viscera [13,14]. In addition, a positive relationship between a history of having cuts on hands and seropositivity has been demonstrated [15].

Information regarding Q fever in Kenya is limited. The disease was first described in people in Nakuru in 1955 [16]. A review of Q fever in Kenya was published in 2016 reporting the seroprevalence in people to range from 3.0 to 35.8% [17]. We have previously conducted a study in a smallholder farming community in western Kenya and reported the prevalence in the general population to be 2.5% (95% CI 1.9%–3.3%) [18]. In the present paper we explore the prevalence and occupational risk factors for exposure to *C. burnetii* in slaughterhouse workers from the same community.

## 2 Methods

### 2.1 Ethical approval

The Kenya Medical Research Institute Ethical Review Committee granted ethical approval for this study (SCC Protocol 2086). Signed informed consent was obtained from every participant.

### 2.2 Study site

**Fig. 1:**
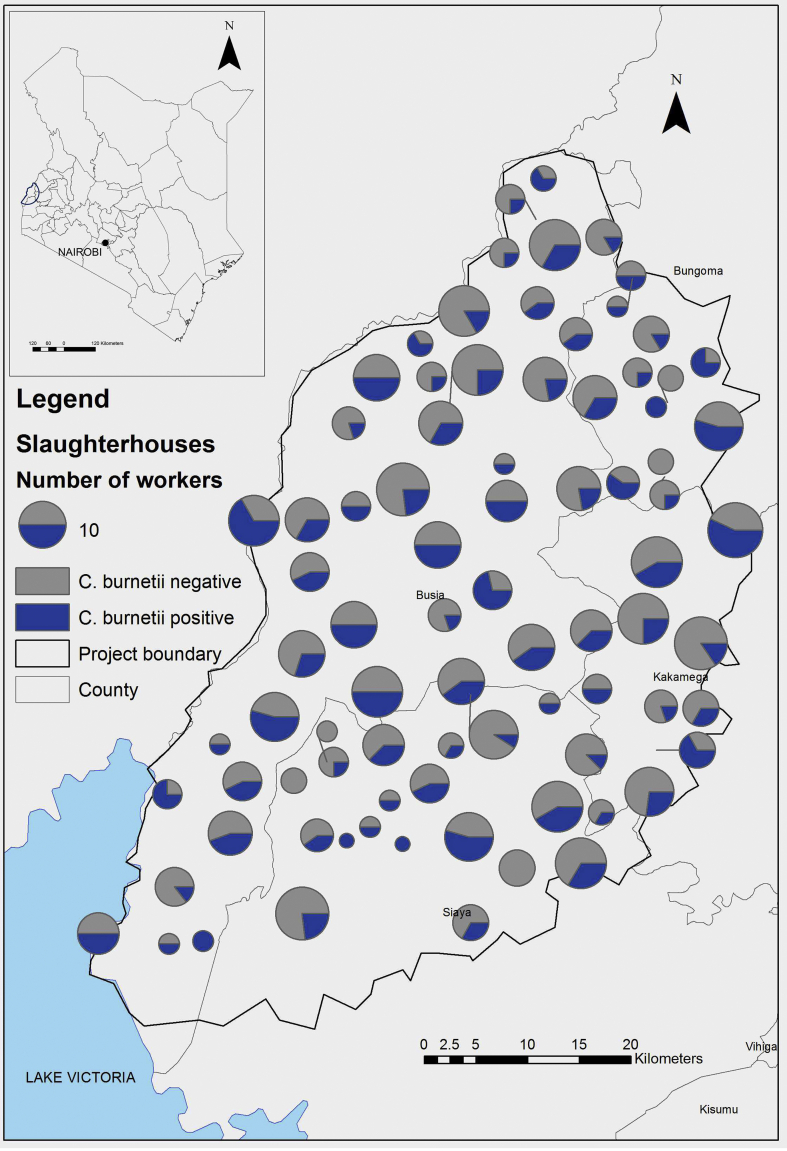
Map of the distribution of *Coxiella burnetii* seropositive and seronegative slaughterhouses. The size of the charts is proportional to the number of workers in each slaughterhouse.

The study area, in western Kenya, was a 45-km radius from Busia town and included Busia County and parts of Bungoma, Siaya and Kakamega Counties (Fig. 1). The region is predominantly rural and the primary livelihood is mixed subsistence farming [19].

### 2.3 Study population and recruitment

The study design has been previously described [20]. In brief, a census of ruminant slaughterhouses (*n* = 88) was conducted between February and November 2012. A handheld GPS device (Garmin eTrex®) was used to georeference slaughterhouses and locations were mapped using ArcGIS™ version 10.2.2 (ESRI, Redlands, California, USA).

Inclusion criteria included workers attending the slaughterhouse on the sampling day and aged over 18 years. Exclusion criteria included being aged greater than 85 years, inebriation, third trimester pregnancy, and extreme aggression towards the project team. The recruitment process has been previously described with up to 12 workers randomly selected from each slaughterhouse [21].

### 2.4 Data and sample collection

A Palm operating system (Palm OS) Personal digital assistant (PDA) using Pendragon Forms 5.1 (Pendragon Software Corporation, Libertyville, IL) was used for data collection. Information was collected regarding the demographics of the workers, risk behaviours, exposure to livestock, roles in the slaughterhouse, personal protective measures practiced at the slaughterhouse and any illness in the previous 12 months. There were two types of workers: those who slaughtered cattle only; and those who slaughtered cattle, goats and sheep. The roles in the slaughterhouse included: the foreman who oversaw the work; the slaughterman who cut the animals throats; the flayer who skinned, eviscerated and sectioned the carcass; the person who cleaned the intestines; and the person who cleaned the slaughterhouse. These are distinct roles in slaughterhouses in western Kenya with limited overlap [21]. Information was also collected at the slaughterhouse level regarding the number and types of animals slaughtered, whether workers wore protective clothing, and the presence of a place for handwashing.

Blood was collected from each participant into 10 ml plain BD Vacutainers® using a 21G or 23G BD Vacutainer® Safetylok™ blood collection set by a clinical officer.

### 2.5 Laboratory analysis

Serum samples were screened for antibodies to *C. burnetii* using the Serion ELISA Classic *C. burnetii* Phase 2 IgG kit (Virion/Serion, Würzburg, Germany) as previously described [22]. Inter-assay variability was reduced by applying a correction factor, calculated by dividing the reference optical density (OD) of the standard serum with the current OD of the standard serum.

$$\mathrm{Correction factor}=\frac{{\mathrm{OD}}_{\mathrm{reference values of the standard serum}}}{{\mathrm{OD}}_{\mathrm{current value of standard serum}}}$$

The measured values of samples were multiplied by the correction factor. Calculation of antibody concentrations was carried out using the logistic-log-model provided by the manufacturer and compared to a batch specific standard curve.

The ELISA used in this study is recommended for serological surveillance [22]. However, previous work demonstrated that only high antibody levels are detected with the manufacturer's recommended cut-off (greater than 30 U/ml) [23]. Since antibodies decrease over time a reduction in cut-off is required to detect low antibodies in seroprevalence studies [8,23], hence we made an adjustment to the cut-off to avoid underestimation of the number of *C. burnetii* seropositive cases in this population [23]. Antibody concentrations greater than 10 U/ml were considered positive [23].

### 2.6 Prevalence estimation

The apparent and true prevalence estimates were calculated using a Bayesian estimation implemented using the *truePrev* function in the *prevalence* package [24] of R (http://CRAN.R-project.org/). The sensitivity and specificity of the ELISA, used to calculate the true prevalence, were 96.4% and 90.2% respectively [23]. Adjustment accounting for the study design was done using sampling weights, calculated by dividing the number of expected workers by the number sampled in each slaughterhouse. This was implemented using the *svydesign* procedure in the *Survey* [25] package in R*.*

The prevalence of antibodies to *C. burnetii* in the general population was estimated from a subset (male and aged over 20 years) of samples (*n* = 409) collected in a concurrent community based study [18] using the adjusted cut-off [23]. The previous study used the same commercial ELISA but with the manufacturer's recommended cut-off. The seroprevalence estimates in the two populations were compared using a non-parametric, chi-square test [26].

Evidence of clustering of the *C. burnetii* seropositive slaughterhouses, defined as a slaughterhouse with at least one positive worker, was determined using the spatial scan statistic [27]. A Bernoulli model was used with 999 iterations in SatScan version 9.0 and a cluster size up to a maximum of 50% of observations (www.satscan.org).

### 2.7 Logistic regression model

Risk factors for *C. burnetii* seropositivity in slaughterhouse workers were identified using multivariable mixed effects (multi-level) logistic regression models. Univariable logistic regression was used to screen variables against pathogen exposure at the individual level. For multicategory variables a likelihood ratio chi-square test was performed by comparing the univariable model to a null model using the *drop1* function in R [28]. Variables examined were those that had previously been identified as risk factors for *C. burnetii* in other settings and included sex, age, animal contact, food consumption practices, use of protective clothing, the species and numbers of animals slaughtered, the time worked in the slaughterhouse, and an individual's role within the slaughterhouse. The continuous variables, age, and time worked in the slaughterhouse were examined linearly and in quartiles to explore the potential for non-linear relationships between age or time in the slaughterhouse and the log odds of *Coxiella* seropositivity. The specification of the variables age and time in the slaughterhouse that resulted in the lowest Akaike's second-order information criterion (AIC) were used in the final model. The number of animals slaughtered was log transformed to give a normal distribution.

A multivariable logistic regression model was developed with variables with a *p*-value <0.1 in the univariable analysis. Correlation analysis was performed by calculating the phi coefficient for categorical variables in the *psych* package [29] and Pearson's correlation coefficient for continuous variables using the *cor.test* function in R [30]. Variables were considered correlated if the coefficient was >0.5 and the variable with the highest *p*-value during univariable logistic regression analysis was excluded. Models were developed using the *glmer* function in the *lme4* package [28] and included a random effect to account for the clustering of the workers within slaughterhouses. Age and gender were included in the model as potential confounders. Model selection was conducted using a backwards-stepwise approach, starting with a full model with all predictors and then dropping variables with the highest *p*-value in a stepwise fashion until the model with the lowest AIC was identified. We compared model performance with different interaction terms (eg. gender and job in the slaughterhouse, gender and animal type). Variance Inflation Factors (VIFs) were calculated to check for collinearity using the *vif* function in the package *car* [31]. Variables were removed from the model if VIFS >4.

A multivariable mixed effects logistic regression model was used to measure the relationship between *C. burnetii* seropositivity in slaughterhouse workers and having pneumonia in the previous year. Age and gender were included in the model as potential confounders.

## 3 Results

We recruited 566 slaughterhouse workers from 84 ruminant slaughterhouses. Four slaughterhouses declined to participate in the study. Two hundred and ten workers were seropositive for *C. burnetii* with an apparent prevalence of 37.1% (95% Confidence Interval (CI) 33.2–41.2%). The adjusted prevalence accounting for the design effect was 38.9% (95% CI 34.8–42.9%). The true prevalence was 31.8% (95% CI 27.2–36.4%) after adjustment for the sensitivity and specificity of the test. The seroprevalence in the community sample among males aged more than 20 years old was 18.8% (95% CI 15.4–22.9%). The true prevalence in the community sample was 10.6% (95% CI 6.4–15.2%) after adjustment for the sensitivity and specificity of the test. There was a statistically significant difference between the prevalence in the slaughterhouse workers and the age and sex matched community sample (Chi^2^ = 38.3, df = 1, *p* < 0.001).

Seropositive slaughterhouse workers were identified in 78 (92.9%) slaughterhouses which were distributed throughout the study area (Fig. 1). There was no evidence of significant spatial clustering of slaughterhouses with seropositive workers. One cluster was detected containing 36 slaughterhouses and the relative risk (RR) of slaughterhouses inside the cluster having a seropositive worker compared to outside was 1.17 (*p*-value =0.244).

The majority of workers were male (96.6%) and a higher proportion of male workers were seropositive for *C. burnetii* than female workers, with only 3 of the 19 female workers seropositive (OR 3.25; 95% CI 0.93–11.28). The range in age of slaughterhouse workers was 18–82 years with the mean age 41 years. The time worked in the slaughterhouse ranged from 0 to 59 years with a mean time of 10 years. There was no significant relationship between age or time worked in the slaughterhouse and seropositivity.

There were more workers involved with cattle slaughter (*n* = 370) but workers who slaughtered all ruminants (*n* = 184) were more likely to be seropositive than those slaughtering cattle only (OR 1.48; 95% CI 1.03–2.13). There was a significant association between seropositivity and the role in the slaughterhouse (Table 1). The number of animals slaughtered per week by an individual worker ranged from 1 to 75 with a mean of 6. There was not a significant relationship between the number of animals slaughtered and seropositivity. There were no significant associations between *C. burnetii* seropositivity and contact with ruminants outside the slaughterhouse or with food consumption practices such as consuming raw milk or blood (Table 1). Wearing protective clothing or boots was not associated with *C. burnetii* seropositivity (Table 1). There were also no associations between hygiene practices in the slaughterhouses (handwashing facilities, workers wearing protective clothing) and *C. burnetii* seropositivity in workers (Table 2).

**Table 1:** Results of univariable analysis for individual level risk factors for seropositivity to *C. burnetii* in slaughterhouse workers from western Kenya.

Variable	Number (%) n = 566	*C. burnetii* seropositive (%) *n* = 210	Odds Ratio (95% CI)	p-value
Gender				
Male	547 (96.6)	207 (37.8)	3.25 (0.93–11.28)	0.064
Female	19 (3.4)	3 (15.7)	1	
Age (quartiles)				
18–29 years	128 (22.6)	48 (37.5)	1	0.764^a^
30–37 years	143 (25.3)	49 (34.3)	0.87 (0.53–1.43)	
38–51 years	151 (26.7)	55 (36.4)	0.95 (0.59–1.56)	
52–82 years	144 (25.4)	58 (40.3)	1.12 (0.69–1.83)	
Age (linear)			1.00 (0.99–1.02)	0.435^b^
Cattle contact				
Yes	417 (73.7)	151(36.2)	0.87 (0.59–1.27)	0.463
No	149 (26.3)	59 (39.6)	1	
Sheep contact				
Yes	153 (27.0)	60 (36.8)	1.13 (0.77–1.66)	0.527
No	413 (73.0)	150 (36.3)	1	
Goat contact				
Yes	244(43.1)	86 (35.2)	0.87 (0.62–1.23)	0.426
No	322 (56.9)	124 (38.5)	1	
Wearing an apron/dust coat				
Always	335 (59.2)	125 (37.3)	1	0.360
Sometimes	101 (17.8)	32 (31.7)	0.78 (0.48–1.25)	
Never	130 (23.0)	53 (40.7)	1.16 (0.76–1.75)	
Wearing boots				
Always	306 (54.0)	110 (35.9)	0.90 (0.64–1.26)	0.537
Never	260 (46.0)	100 (38.5)	1	
Drinking raw milk				
Yes	20 (3.5)	8 (40.0)	0.97 (0.92–1.03)	0.382
No	546 (96.6)	202 (37.0)	1	
Consuming animal blood				
Yes	323 (57.1)	126 (39.0)	1.21 (0.86–1.70)	0.279
No	243 (42.9)	84 (34.6)	1	
Time worked in the slaughterhouse (quartiles)				
Less than 4 years	164 (29.0)	53 (32.3)	1	0.035^c^
5–7 years	137 (24.2)	59 (43.1)	1.58 (0.99–2.54)	
8–14 years	129 (22.7)	39 (30.2)	0.91 (0.55–1.49)	
15–59 years	136 (24.0)	59 (43.3)	1.60 (1.00–2.57)	
Time worked in the slaughterhouse (linear)			1.02 (1.00–1.03)	0.054^d^
Number of animals slaughtered per week (individual) (log_10_)			1.02 (0.56–1.86)	0.944
Species slaughtered by individuals				
Cattle only	370 (65.4)	127 (34.3)	1	
Cattle, goats and sheep	196 (34.6)	83 (42.3)	1.41 (0.99–2.01)	0.061
Job in the slaughterhouse				
Slaughterman or foreman	76 (13.4)	17 (22.4)	1	0.033
Cleaner	26 (4.6)	10 (38.5)	2.17 (0.83–5.65)	
Cleans the intestines	42 (7.4)	16 (38.1)	2.14 (0.94–4.87)	
Flayers	422 (74.6)	167 (39.6)	2.27 (1.28–4.03)	

a AIC = 755.4.

b AIC = 751.9.

c AIC = 747.9.

d AIC = 748.8.

**Table 2:** Results of univariable analysis examining slaughterhouse level risk factors for seropositivity to *C. burnetii* in slaughterhouse workers from western Kenya.

Variable	Number (%) n = 566	*C. burnetii* seropositive (%) *n* = 210	Odds Ratio (95% CI)	p- value
Apron worn by workers				
Always	283 (50.0)	109 (38.5)	1	0.560
Sometimes	218 (38.5)	75 (34.4)	0.84 (0.58–1.21)	
Never	65 (11.5)	26 (40.0)	1.06 (0.61–1.85)	

Boots worn by workers				
Always	317 (56.0)	117 (36.9)	1	
Sometimes	147 (26.0)	56 (38.0)	1.05 (0.70–1.57)	0.806
Never	102 (18.0)	37 (36.2)	0.97 (0.61–1.55)	0.908

Place for handwashing				
Yes	184 (32.5)	68 (37.0)	1.03 (0.71–1.48)	0.892
No	382 (67.5)	141 (36.9)	1	

Species slaughtered (slaughterhouse)				
Cattle only	292 (52.6)	98 (33.6)	0.73 (0.52–1.03)	0.072
Cattle, goats and sheep	274 (48.4)	112 (40.9)	1	
Number of animals slaughtered per week (slaughterhouse) (log_10_)			1.11 (0.76–1.62)	0.579

### 3.1 Multivariable logistic regression

The final multivariable model for *C. burnetii* seropositivity in individual slaughterhouse workers is shown in Table 3. The final model included: age (linear), gender, job in the slaughterhouse, and species slaughtered by individuals. The variable, species slaughtered at the slaughterhouse level, was not included in the model since it was correlated with species slaughtered by individuals (phi = 0.66). Age was included as a potential confounder. There were no significant interactions. Risk factors for exposure to *C. burnetii* were: being male, OR 5.40 (95% CI 1.38–21.22); slaughtering cattle, goats and sheep, OR 1.52 (95% CI 1.06–2.19); and cleaning the slaughterhouse, OR 3.98 (95% CI 1.39–11.43), cleaning the intestines, OR 3.24 (95% CI 1.36–7.73) and flaying the carcass OR 2.63 (95% CI 1.46–4.75) compared to the slaughterman or foreman.

**Table 3:** Results of the multivariable analysis for *C. burnetii* seropositivity in slaughterhouse workers.

Variables	OR (95% CI)	*p–*value	VIFs
Gender			
Female	1		
Male	5.40 (1.38–21.22)	0.016	1.194
Age (Linear)	1.01 (1.00–1.02)	0.129	1.054
Species slaughtered by individuals			
Cattle only	1		
Cattle, goats and sheep	1.52 (1.06–2.19)	0.024	1.018
Job in the slaughterhouse			
Slaughterman and foreman	1		1.254
Cleaner	3.98 (1.39–11.43)	0.010	
Cleans intestines	3.24 (1.36–7.73)	0.008	
Flayer	2.63 (1.46–4.75)	0.001	

### 3.2 Clinical symptoms

There was a positive but non-significant association between workers who reported having pneumonia in the previous 12 months and seropositivity (n = 8/13; OR 2.58, 95% CI 0.82–8.08).

## 4 Discussion

The apparent seroprevalence for antibodies to *C. burnetii* in slaughterhouse workers in western Kenya was 37.1% (95% CI 33.2–41.2%), the prevalence when adjusted for the estimated sensitivity and specificity of the ELISA was 31.8% (95% CI 27.2–36.4%) and for the study design was 38.9% (95% CI 34.8–42.9%). Previous reports have estimated seroprevalence of Q fever in slaughterhouse workers to range from 5 to 90% [12,14,15,[32], [33], [34], [35]]. It is difficult to compare our results with reports from different regions because different diagnostic tests have been used [8]. A review conducted in 2018 determined the pooled estimate of seroprevalence for *C. burnetii* in slaughterhouse workers to be 26% (95% CI 18–35%) independent of age or time worked in the slaughterhouse, which is consistent with our findings [36].

The *C. burnetii* seroprevalence in a concurrent community based sample in the same study area was 2.5% (95% CI 1.9–3.3%) [18]. The previous study used the same commercial ELISA but with the manufacturer's recommended cut-off. After adjusting the analysis using an updated cut-off recommended for serosurveys [23] and using a gender and age-matched sample, the seroprevalence estimate was 18.8% (95% CI 15.4–22.9%). The difference between the seroprevalence in slaughterhouse workers and the community suggests that workers are significantly more exposed to *C. burnetii* than the equivalent age-group in the male population. This is consistent with other studies that have demonstrated a higher seroprevalence in slaughterhouse workers than other occupation groups [12,32,34].

There was no evidence for spatial clustering, with *C. burnetii* seropositive workers distributed throughout the study area. The risk areas for *C. burnetii* seropositive cattle were reported to be the central north and south-east of the study area [18] but there are no reports of seroprevalence in small ruminants. It is likely that animals for slaughter are moved around after purchase by traders or butchers [37]. Workers who slaughtered cattle, goats and sheep were more likely to be seropositive than workers who slaughter only cattle, which may suggest a higher transmission risk from goats and sheep in the study area. Further investigation of the prevalence of *C. burnetii* exposure in small ruminants in this region is recommended since studies in other regions of Kenya have indicated a high seroprevalence in these species [38].

The position or job in the slaughterhouse has previously been identified as a risk factor for *C. burnetii* seropositivity and this was demonstrated in our study [[12], [13], [14]]. The workers with the least contact with the carcass (slaughterman and foreman) had the lowest risk of being *C. burnetii* seropositive compared to workers with contact with the viscera and fluids (flayers and cleaners of intestines). Flaying and contact with the viscera have been reported as risk factors in previous studies [14,39,40]. While this occupational exposure is likely to put slaughterhouse workers at particularly elevated risk for Q-fever, 15% of homesteads in the study area also reported slaughtering animals at home which may represent a risk for *C. burnetii* exposure among livestock keepers in this region [19]. Previous studies have reported that male workers are more likely to be seropositive for *C. burnetii* [11,13] which is consistent with our results and may be related to the different roles in the slaughterhouse with men conducting high risk activities such as flaying and eviscerating [14,40].

This study investigating risk factors for *C. burnetii* seropositivity in slaughterhouse workers is the first of its type in Kenya and adds to the global literature on Q-fever epidemiology, filling an important gap in knowledge in the East African region. Slaughterhouse workers are a high-risk group for exposure to *C. burnetii*. Health care workers should be trained to recognise zoonotic diseases in occupationally exposed groups as early treatment can prevent Q fever from becoming chronic. However, the non-specific clinical presentation of Q fever may delay definitive diagnosis. Point of care tests may improve the accuracy of diagnosis but further information is required on the incidence of Q fever to determine if point of care tests are an appropriate and affordable means of detecting disease in this region.

Slaughterhouse workers should be educated about the risks and ways to prevent or reduce transmission of zoonotic diseases. Simple hygiene measures such as washing hands, covering wounds and wearing protective equipment may reduce exposure [41]. Workers were not seen to wear masks or gloves at the time of this research [21]. However, since the COVID-19 pandemic these items may be more accessible, affordable and acceptable to workers [42]. Vaccination is an alternative control measure and is advocated for at-risk occupational groups in Australia. However, the vaccine can result in severe reactions in previously exposed people which limits its use in a high prevalence setting [43]. There were a small number of workers (*n* = 13) who reported having pneumonia in the previous 12 months of which 62% were seropositive. However, the contribution of *C. burnetii* as a cause of pneumonia in these workers cannot be determined from our study. A better understanding regarding the incidence and burden of acute and chronic Q fever in the region is required to inform a cost benefit analysis to determine if resources should be focused on intervention programs for this zoonotic disease.

## Declaration of interests

The authors declare that they have no known competing financial interests or personal relationships that could have appeared to influence the work reported in this paper.

## Authors' contribution

EAJC designed and implemented the study. EMF, BMdeCB and SK assisted with study design. LFT, and WAdG, assisted with implementation of the study. VK and AK conducted laboratory analysis. EAJC, BMdCB and WAdG undertook data analysis. EMF obtained funding for the study. All authors made contributions to conception, design, and revision of the manuscript. All authors read and approved the final manuscript.
